# Correction: Evaluation of collapsible deformation of foundation under rectangular load based on the improved binary medium model

**DOI:** 10.1371/journal.pone.0336044

**Published:** 2025-11-03

**Authors:** Nadeem Abbas, Muhammad Akbar, S.B.A. Elsayed, Gehan Ahmed, Ahmed M. Yosri, Muhammad Usman Arshid, Mahmoud Elkady

There is an error in affiliation 4 for author S.B.A. Elsayed. The correct affiliation 4 is: Department of Mathematics, College of Science, Jouf University, P.O. Box 2014, Sakaka, Jouf, Saudi Arabia.
